# Decreased Influenza Activity During the COVID-19 Pandemic — United States, Australia, Chile, and South Africa, 2020

**DOI:** 10.15585/mmwr.mm6937a6

**Published:** 2020-09-18

**Authors:** Sonja J. Olsen, Eduardo Azziz-Baumgartner, Alicia P. Budd, Lynnette Brammer, Sheena Sullivan, Rodrigo Fasce Pineda, Cheryl Cohen, Alicia M. Fry

**Affiliations:** ^1^Influenza Division, National Center for Immunization and Respiratory Diseases, CDC; ^2^World Health Organization Collaborating Centre for Reference and Research on Influenza, Royal Melbourne Hospital, and Doherty Department, University of Melbourne, at the Peter Doherty Institute for Infection and Immunity, Melbourne, Australia; ^3^Virology Department, Public Health Institute of Chile, Santiago, Chile; ^4^Center for Respiratory Diseases and Meningitis, National Institute for Communicable Diseases, Johannesburg, South Africa; ^5^School of Public Health, Faculty of Health Sciences, University of the Witwatersrand, Johannesburg, South Africa.

After recognition of widespread community transmission of SARS-CoV-2, the virus that causes coronavirus disease 2019 (COVID-19), by mid- to late February 2020, indicators of influenza activity began to decline in the Northern Hemisphere. These changes were attributed to both artifactual changes related to declines in routine health seeking for respiratory illness as well as real changes in influenza virus circulation because of widespread implementation of measures to mitigate transmission of SARS-CoV-2. Data from clinical laboratories in the United States indicated a 61% decrease in the number of specimens submitted (from a median of 49,696 per week during September 29, 2019–February 29, 2020, to 19,537 during March 1–May 16, 2020) and a 98% decrease in influenza activity as measured by percentage of submitted specimens testing positive (from a median of 19.34% to 0.33%). Interseasonal (i.e., summer) circulation of influenza in the United States (May 17–August 8, 2020) is currently at historical lows (median = 0.20% tests positive in 2020 versus 2.35% in 2019, 1.04% in 2018, and 2.36% in 2017). Influenza data reported to the World Health Organization’s (WHO’s) FluNet platform from three Southern Hemisphere countries that serve as robust sentinel sites for influenza from Oceania (Australia), South America (Chile), and Southern Africa (South Africa) showed very low influenza activity during June–August 2020, the months that constitute the typical Southern Hemisphere influenza season. In countries or jurisdictions where extensive community mitigation measures are maintained (e.g., face masks, social distancing, school closures, and teleworking), those locations might have little influenza circulation during the upcoming 2020–21 Northern Hemisphere influenza season. The use of community mitigation measures for the COVID-19 pandemic, plus influenza vaccination, are likely to be effective in reducing the incidence and impact of influenza, and some of these mitigation measures could have a role in preventing influenza in future seasons. However, given the novelty of the COVID-19 pandemic and the uncertainty of continued community mitigation measures, it is important to plan for seasonal influenza circulation in the United States this fall and winter. Influenza vaccination of all persons aged ≥6 months remains the best method for influenza prevention and is especially important this season when SARS-CoV-2 and influenza virus might cocirculate (*1*).

Data from approximately 300 U.S. clinical laboratories located throughout all 50 states, Puerto Rico, Guam, and the District of Columbia that participate in virologic surveillance for influenza through either the U.S. WHO Collaborating Laboratories System or the National Respiratory and Enteric Virus Surveillance System* were used for this analysis. Clinical laboratories primarily test respiratory specimens for diagnostic purposes, and data from these laboratories provide useful information on the timing and intensity of influenza activity. The median number of specimens tested per week and the median percentage of samples testing positive for influenza during September 29, 2019–February 29, 2020 (surveillance weeks 40–9, the period before the March 1, 2020 declaration of a national emergency related to COVID-19^†^) were compared with those tested during March 1–May 16, 2020 (weeks 10–20 after the declaration); data from three previous influenza seasons are presented as a comparison. To assess influenza virus activity in the Southern Hemisphere, influenza laboratory data from clinical and surveillance platforms reported from Australia, Chile, and South Africa to WHO’s FluNet^§^ platform were analyzed. For each country, the percentage of samples testing positive for influenza for April–July (weeks 14–31) for four seasons (2017–2020) are presented. Selected measures implemented to respond to COVID-19 in these countries were ascertained from government websites. All data used were in the public domain.

In the United States, influenza activity (measured by percentage of respiratory specimens submitted for influenza testing that yielded positive results) began to increase in early November 2019, and >20% of specimens were positive during December 15, 2019–March 7, 2020 (weeks 51–10), after which activity declined sharply (Figure 1). Percent positivity peaked on week 6 at 30.25% and decreased 14.90% by week 9, compared with an 89.77% decrease during weeks 10–13. By the week of March 22, 2020 (week 13), when the number of samples tested remained very high, percent positivity dropped to 2.3%, and since the week of April 5, 2020 (week 15), has remained <1%. The median number of specimens tested for influenza each week decreased from 49,696 during September 29, 2019–February 29, 2020 (weeks 40–9), to 19,537 during March 1–May 16, 2020 (weeks 10–20), representing a 61% decrease. During these same two periods, influenza activity decreased 98%, from a median of 19.34% to 0.33% of submitted respiratory specimens testing positive for influenza. Interseasonal circulation of influenza in the United States (May 17–August 8, 2020; weeks 21–32) is now at historical lows (weekly median 0.20% of samples testing positive in 2020 versus 2.35% in 2019, 1.04% in 2018 and 2.36% in 2017).

**FIGURE 1:**
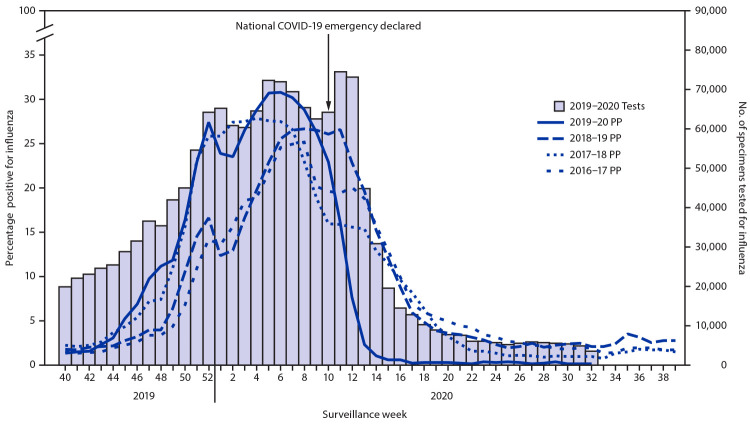
Number of respiratory specimens tested and percentage testing positive for influenza, by year — United States, 2016–17 through 2019–20 seasons **Source:** FluView Interactive. https://www.cdc.gov/flu/weekly/fluviewinteractive.htm. **Abbreviation:** PP = percentage positive.

In the Southern Hemisphere countries of Australia, Chile, and South Africa, only 33 influenza positive test results were detected among 60,031 specimens tested in Australia, 12 among 21,178 specimens tested in Chile, and six among 2,098 specimens tested in South Africa, for a total of 51 influenza positive specimens (0.06%, 95% confidence interval [CI] = 0.04%–0.08%) among 83,307 tested in these three countries during April–July 2020 (weeks 14–31). In contrast, during April–July in 2017–2019, 24,512 specimens tested positive for influenza (13.7%, 95% CI = 13.6%–13.9%) among 178,690 tested in these three countries (Figure 2).

**FIGURE 2:**
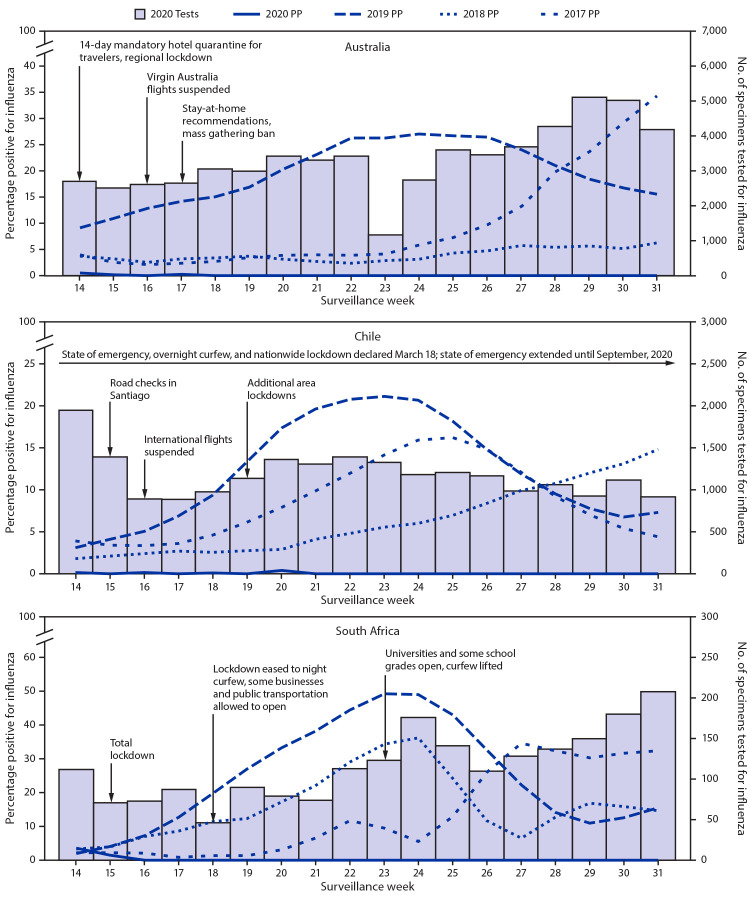
Number of specimens tested and percentage testing positive for influenza, by year — Australia, Chile, and South Africa, April–August (weeks 14–31), 2017–20 **Source:** FluNet. https://www.who.int/influenza/gisrs_laboratory/flunet/en/. **Abbreviation:** PP = percentage positive.

In the United States, the COVID-19 national emergency was declared on March 1, 2020, but states began implementing a range of COVID-19 mitigation measures in late February, including school closures, bans on mass gatherings, and stay-at-home orders (*2*). In addition, some emphasis was placed on individual measures, such as mask wearing, staying home while sick, and social distancing. In Australia, a 14-day mandatory hotel quarantine was introduced for all returned travelers on March 29; regional lockdowns began in early April, followed by a stay-at-home recommendation and bans on gatherings in mid-April. Some easing of measures began in late April.^¶^ In Chile, the president declared a state of emergency on March 18, which remains in effect into September. In addition, in mid-March an overnight curfew and a nationwide lockdown were implemented. Since then, the lockdown has been lifted regionally, based on disease activity; however, recommendations to stay at home and socially distance, as well as mandatory use of masks are all still in place.** In South Africa, a total lockdown was imposed on April 9, with some easing of measures starting on May 1.^††^ The community mitigation strategies implemented to prevent the spread of COVID-19, including both community and individual-level measures, appear to have substantially reduced transmission of influenza in all these countries.

## Discussion

In the United States, influenza virus circulation declined sharply within 2 weeks of the COVID-19 emergency declaration and widespread implementation of community mitigation measures, including school closures, social distancing, and mask wearing, although the exact timing varied by location (*2*). The decline in influenza virus circulation observed in the United States also occurred in other Northern Hemisphere countries (*3*,*4*) and the tropics (*5*,*6*), and the Southern Hemisphere temperate climates have had virtually no influenza circulation. Although causality cannot be inferred from these ecological comparisons, the consistent trends over time and place are compelling and biologically plausible. Like SARS-CoV-2, influenza viruses are spread primarily by droplet transmission; the lower transmissibility of seasonal influenza virus (R_0_ = 1.28) compared with that of SARS-CoV-2 (R_0_ = 2–3.5) (*7*) likely contributed to a more substantial interruption in influenza transmission. These findings suggest that certain community mitigation measures might be useful adjuncts to influenza vaccination during influenza seasons, particularly for populations at highest risk for developing severe disease or complications.

Initially, declines in influenza virus activity were attributed to decreased testing, because persons with respiratory symptoms were often preferentially referred for SARS-CoV-2 assessment and testing. However, renewed efforts by public health officials and clinicians to test samples for influenza resulted in adequate numbers tested and detection of little to no influenza virus. Further, some countries, such as Australia, had less stringent criteria for testing respiratory specimens than in previous seasons and tested markedly more specimens for influenza but still detected few with positive results during months when Southern Hemisphere influenza epidemics typically peak. A new Food and Drug Administration–approved multiplex diagnostic assay for detection of both SARS-CoV-2 and influenza viruses could improve future surveillance efforts (https://www.cdc.gov/coronavirus/2019-ncov/lab/multiplex.html).

It is difficult to separate the effect that individual community mitigation measures might have had on influenza transmission this season. Although school-aged children can drive the spread of influenza, the effectiveness of school closures alone is not clear because adults have other exposures (*8*). There is evidence to support the use of face masks by infected persons to reduce transmission of viral respiratory illnesses to others and growing evidence to support their use (in the health care setting, in households, and in the community) to protect the healthy wearer from acquiring infection. More data are needed to assess effectiveness of different types of masks in different settings (*9*). Data from the current pandemic might help answer critical questions about the effect of community mitigation measures on transmission of influenza or other respiratory diseases. In addition, assessing acceptability of effective measures would be critical, because acceptability is likely to be inversely correlated with the stringency of the measure.

The findings in this report are subject to at least four limitations. First, an ecologic analysis cannot demonstrate causality, although the consistency of findings across multiple countries is compelling. Second, other factors, such as the sharp reductions in global travel or increased vaccine use, might have played a role in decreasing influenza spread; however, these were not assessed. Third, viral interference might help explain the lack of influenza during a pandemic caused by another respiratory virus that might outcompete influenza in the respiratory tract (*10*). This possibility is less likely in the United States because influenza activity was already decreasing before SARS-CoV-2 community transmission was widespread in most parts of the nation. Finally, it is possible that the declines observed in the United States were just the natural end to the influenza season. However, the change in the decrease percent positivity after March 1 was dramatic, suggesting other factors were at play.

The global decline in influenza virus circulation appears to be real and concurrent with the COVID-19 pandemic and its associated community mitigation measures. Influenza virus circulation continues to be monitored to determine if the low activity levels persist after community mitigation measures are eased. If extensive community mitigation measures continue throughout the fall, influenza activity in the United States might remain low and the season might be blunted or delayed. In the future, some of these community mitigation measures could be implemented during influenza epidemics to reduce transmission, particularly in populations at highest risk for developing severe disease or complications. However, in light of the novelty of the COVID-19 pandemic and the uncertainty of continued community mitigation measures, it is important to plan for seasonal influenza circulation this fall and winter. Influenza vaccination for all persons aged ≥6 months remains the best method for influenza prevention and is especially important this season when SARS-CoV-2 and influenza virus might cocirculate *(**1**)*.

**Summary**

**What is already known about this topic?**

Influenza activity is currently low in the United States and globally.

**What is added by this report?**

Following widespread adoption of community mitigation measures to reduce transmission of SARS-CoV-2, the virus that causes COVID-19, the percentage of U.S. respiratory specimens submitted for influenza testing that tested positive decreased from >20% to 2.3% and has remained at historically low interseasonal levels (0.2% versus 1–2%). Data from Southern Hemisphere countries also indicate little influenza activity.

**What are the implications for public health practice?**

Interventions aimed against SARS-CoV-2 transmission, plus influenza vaccination, could substantially reduce influenza incidence and impact in the 2020–21 Northern Hemisphere season. Some mitigation measures might have a role in reducing transmission in future influenza seasons.

## Footnotes

* https://www.cdc.gov/flu/weekly/overview.htm.

†
https://www.whitehouse.gov/presidential-actions/proclamation-declaring-national-emergency-concerning-novel-coronavirus-disease-covid-19-outbreak/ .

§
https://www.who.int/influenza/gisrs_laboratory/flunet/en/ .

¶
https://www.health.gov.au/news/health-alerts/novel-coronavirus-2019-ncov-health-alert/coronavirus-covid-19-restrictions .

**
https://www.gob.cl/coronavirus/plandeaccion .

††
https://sacoronavirus.co.za/ ; https://www.gov.za/coronavirus/alert-level-2 .
